# PP20 Rituximab Biosimilar Compared With Reference In Non-Hodgkin’s Lymphoma: Results From A Cochrane Review

**DOI:** 10.1017/S0266462325102407

**Published:** 2025-12-29

**Authors:** Annemeri Livinalli, Tais F. Galvao, Luciane C. Lopes, Ivan Zimmermann, Marcus T. Silva

## Abstract

**Introduction:**

Rituximab is a monoclonal antibody used to treat non-Hodgkin’s lymphoma. Since the expiration of the reference rituximab’s patent, several biosimilars have been marketed. As biosimilar products are not identical to reference medicines, synthesis of evidence comparing them is needed to understand their effects and harms.

**Methods:**

We performed a Cochrane systematic review and searched main bibliographic (CENTRAL, MEDLINE, Embase, Web of Science) and clinical trials databases up to February 2024. We included head-to-head randomized controlled trials conducted in adults with lymphoma treated with rituximab biosimilar or reference. When possible, we pooled the results of the following outcomes using random-effects meta-analyses: progression-free survival, duration of response, overall survival, serious adverse events, objective response rate, and health-related quality of life. We used the revised Cochrane risk-of-bias tool to assess the risk of bias and GRADE to evaluate the certainty of evidence of critical and important outcomes.

**Results:**

We included 16 studies (n=4,342 participants). The overall risk of bias was low. Rituximab biosimilar was likely similar to reference in progression-free survival, duration of response, and overall survival (moderate certainty evidence; data not pooled as survival estimates were adjusted for different factors or reported as rates); serious adverse events (risk ratio [RR] 1.03, 95% confidence interval [CI]: 0.94, 1.14]; 15 studies, n=4,197; moderate certainty evidence); objective response (RR 1.01, 95% CI: 0.98, 1.04; 15 studies, n=3,852; moderate certainty evidence); and mortality (RR 0.97, 95% CI: 0.70, 1.35; 8 studies, n=2,557; high certainty evidence). Imprecision was the main reason for downgrading certainty in the findings.

**Conclusions:**

Treatment with rituximab biosimilars was likely similar to the reference in survival outcomes and rates of adverse events. The overall certainty of evidence was moderate.

